# Study of Beryllium, Magnesium, and Spodium Bonds to Carbenes and Carbodiphosphoranes

**DOI:** 10.3390/molecules26082275

**Published:** 2021-04-14

**Authors:** Mirosław Jabłoński

**Affiliations:** Faculty of Chemistry, Nicolaus Copernicus University, 87-100 Toruń, Poland; teojab@chem.umk.pl; Tel.: +48-056-611-4695

**Keywords:** beryllium bond, magnesium bond, zinc bond, spodium bond, carbene, carbodiphosphoranes, intermolecular interaction, beryllium, magnesium, zinc

## Abstract

The aim of this article is to present results of theoretical study on the properties of C⋯M bonds, where C is either a carbene or carbodiphosphorane carbon atom and M is an acidic center of MX${}_{2}$ (M = Be, Mg, Zn). Due to the rarity of theoretical data regarding the C⋯Zn bond (i.e., the zinc bond), the main focus is placed on comparing the characteristics of this interaction with C⋯Be (beryllium bond) and C⋯Mg (magnesium bond). For this purpose, theoretical studies (ωB97X-D/6-311++G(2df,2p)) have been performed for a large group of dimers formed by MX${}_{2}$ (X = H, F, Cl, Br, Me) and either a carbene ((NH${}_{2}$)${}_{2}$C, imidazol-2-ylidene, imidazolidin-2-ylidene, tetrahydropyrymid-2-ylidene, cyclopropenylidene) or carbodiphosphorane ((PH${}_{3}$)${}_{2}$C, (NH${}_{3}$)${}_{2}$C) molecule. The investigated dimers are characterized by a very strong charge transfer effect from either the carbene or carbodiphosphorane molecule to the MX${}_{2}$ one. This may even be over six times as strong as in the water dimer. According to the QTAIM and NCI method, the zinc bond is not very different than the beryllium bond, with both featuring a significant covalent contribution. However, the zinc bond should be definitely stronger if delocalization index is considered.

## 1. Introduction

Undoubtedly, one can get an impression that there has been a kind of race that has been going on for over a dozen years related to the introduction of various names for various interatomic contacts. The current situation with this nomenclature has recently been well captured by Alkorta, Elguero, and Frontera in a review article in *Crystals* [1]. Thus, in addition to hydrogen bonds [2,3,4,5,6,7,8,9,10,11,12,13,14,15,16], which have been well established for a hundred years, we also now have alkali bonds [17,18,19,20,21,22], alkaline earth metal bonds [23,24,25,26,27,28,29,30,31,32,33,34], triel bonds [35,36,37,38,39,40,41,42,43,44,45,46], tetrel bonds [47,48,49,50,51,52,53,54,55], pnictogen bonds [55,56,57,58,59,60,61,62,63], chalcogen bonds [64,65,66,67,68,69,70,71,72], halogen bonds [73,74,75,76,77,78], and aerogen (noble gas) bonds [79,80,81]. Apart from the obvious hydrogen bonds, the remaining terms refer consecutively to the interaction in which a Lewis acid is an element of groups 1–2 and then 13–18 of the periodic table. In circulation, there also exist names that refer to individual elements of group 1 or 2, namely lithium bonds [17,18,19,20] and sodium bonds [21,22] in the former case and beryllium bonds [23,24,25,26,27,28,29,30], magnesium bonds [23,29,30,31,32,33], and calcium bonds [34] in the latter. Interactions involving various transition metals have not been called so willingly; however, in the case of interactions in which the Lewis acid center is a metal from groups 10 or 11, the term regium bonds [82,83,84,85,86,87,88,89,90] is relatively common. It is worth mentioning here that for interactions involving metals from group 11, the name coinage-metal bonds was previously introduced. In the aforementioned review article, Alkorta et al. proposed that interactions involving group 12 metals be called spodium bonds [1,91,92,93]. Unfortunately, for the metals of this group, this name seems not very intuitive.

**Figure 1:**
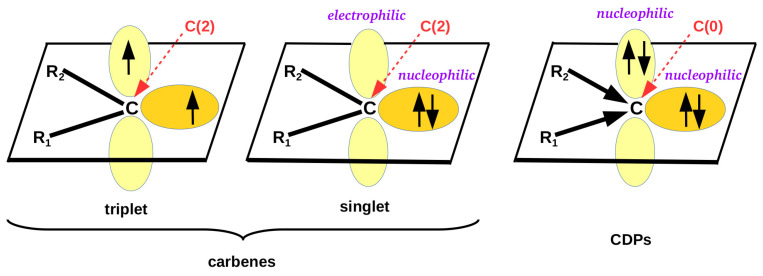
Electronic states of carbenes and carbodiphosphoranes.

Due to their specific electronic structure, carbenes occupy particular position in organic chemistry [94,95,96,97,98,99,100,101,102,103]. This peculiar electronic structure of carbenes results from the fact that the carbene carbon atom is merely divalent and therefore forms only one (C=R) or at most two (CR${}_{1}$R${}_{2}$) covalent bonds. This chemical situation indicates that only two valence electrons are used in the bonds, whereas the other two are unbound. This is turn leads to two possible spin states, triplet and singlet [98] (Figure 1).

In the triplet spin state, both electrons occupy perpendicular *p* orbitals and have the same spins. In the singlet state, both electrons form a lone pair on one of the perpendicular *p* orbitals. Due to the presence of the often readily available electron lone pair, carbenes in the singlet state are good Lewis bases; i.e., they feature strong nucleophilic properties. Indeed, the nucleophilic properties of carbenes are well known and are often used in organic and organometallic synthesis [94,95,96,97,98,99,100,101,102,103]. Consequently, it is known that carbene carbon atoms willingly form various types of interatomic connections, such as hydrogen bonds [104,105,106,107,108,109,110,111], lithium bonds [102,103,112,113,114], beryllium bonds [102,103,115,116,117,118], magnesium bonds [102,103,118,119,120,121], triel bonds [103,122,123,124,125], tetrel bonds [103,126,127,128], pnictogen bonds [103,129,130,131], chalcogen bonds [103,132], halogen bonds [103,133,134,135,136] (in particular to iodine [133,134]), and aerogen bonds [137]. Moreover, carbenes readily form numerous adducts with transition metals [102,119,138,139], significantly enriching the possibilities of designing syntheses in organometallic chemistry. In this case, the N-heterocyclic carbenes (NHC) are of particular importance [98,99,102,139]. In view of the title of this article, it should be mentioned that complexes for heavier transition metals, i.e., from lower rows of the periodic table, are especially common, while examples of carbenes bound to lighter transition metals, e.g., zinc [119,121], are reported much less frequently. In particular, theoretical reports are missing. In the light of the aforementioned proposal of Alkorta et al. [1], the interaction between the carbene carbon atom and the zinc atom should be classified as a spodium bond.

Figure 1 clearly shows that a singlet carbene, in addition to an electron lone pair, also possesses a formally empty *p* orbital perpendicular to the plane of the molecule, leading to the electrophilic properties of a given carbene [140,141,142,143,144,145]. Thus, carbenes can also act like a Lewis acid interacting with good electron density donors, i.e., Lewis bases. On this topic, the interactions of the carbene carbon atom with nitrogen or phosphorus were definitely the most frequently reported [141]. A practical curiosity is that the formation of phosphorus ylides was considered evidence of the presence of an empty *p* orbital on the carbene carbon atom of singlet carbenes [98]. It is worth mentioning here that it has only recently been shown by theoretical methods that singlet carbenes can also interact with a hydridic, i.e., possessing partial negative charge, hydrogen atom of silane, leading to a particular case of a tetrel bond (although this case was announced as a hydride-carbene bond) [143,144,145].

Although Hund’s rule favors the spin triplet state over the singlet one [146], the requirements that invert this relationship, i.e., make the singlet state an electronic ground state, are well known. This may happen if either some appropriate geometric requirements are met [147,148,149] or one or both of the substituents $R_{1}$ and $R_{2}$ are σ-electron-withdrawing or π-electron-donating [149,150,151,152,153]. The latter requirement is met especially in the presence of strongly electronegative atoms with lone electron pairs, such as P, N, O, F, Cl, etc. In this case, the preference for the singlet state results from partial delocalization of the electron charge from electron lone pairs of these atoms to the unfilled *p* orbital on the carbene carbon atom (Figure 1).

Apart from carbenes, an equally important and interesting group of organic compounds is the so-called carbodiphosphoranes (CDPs) and their amine analogues [154,155,156,157,158,159,160,161,162,163,164,165,166,167,168,169,170,171,172]. Their uniqueness in the electronic structure (see Figure 1) is that, unlike the previously described carbenes, in CDPs, none of the four valence electrons of the carbon atom participate in ligand binding, and therefore these electrons remain unbound. Instead of covalent bonds as in carbenes, the carbon atom in CDPs is bound to ligands via donor-acceptor R→C bonds [167]. These non-binding valence carbon electrons form two lone pairs, and not just one as in singlet carbenes. It should therefore be expected that CDPs exhibit greater nucleophilic abilities than singlet carbenes, and moreover, they should be felt not only in the plane of the molecule but also in the direction perpendicular to it.

It is understandable that so far, the vast majority of theoretical studies on beryllium and magnesium bonds have used as Lewis bases small molecules containing either some atoms with good electron-donating properties or π bonds [23,24,25,26,27,29,30,31,32]. On the other hand, reports of systems containing spodium bonds [91,92,93], especially with zinc (they could be called zinc bonds) are very rare [91,93]. It is also quite understandable that the research on carbenes and CDPs is mostly experimental. This is of course due to their huge role in organic and organometallic synthesis, as mentioned earlier. For this reason, beryllium bonds, magnesium bonds, or spodium bonds with the zinc atom as the Lewis acid center (i.e., the zinc bonds) with the participation of either carbenes or CDPs are most often found by crystallographic methods in the solid state. In this case, both the carbene (or the CDP) and the Lewis acid interacting with it are molecules containing many different substituents and functional groups, often of considerable size, which makes the systems themselves also generally bulky.

In order to unite these two thematic areas, this article describes the result of theoretical research on a large group of dimers with a beryllium bond, magnesium bond, or zinc bond between various Lewis acids of the MX${}_{2}$ (where M = Be, Mg, Zn and X = H, F, Cl, Br, Me) type and some fundamental carbenes ((NH${}_{2}$)${}_{2}$C, imidazol-2-ylidene, imidazolidin-2-ylidene, tetrahydropyrymid-2-ylidene, and cyclopropenylidene) and CDPs ((PH${}_{3}$)${}_{2}$C and (NH${}_{3}$)${}_{2}$C) acting as a Lewis base. Therefore, the aim of this article is to present the results of theoretical research on the properties of C⋯M bonds, where C is either a carbene or CDP carbon atom. It should be noted that due to the aforementioned scarcity of reports, in particular theoretical ones, on systems featuring a zinc bond (i.e., the spodium bond [1] with the participation of a zinc atom acting as a Lewis acid center), the reported studies on the C⋯Zn bond-containing systems investigated here represent an especially considerable novelty. At the same time, the presented results on the properties of this bond and slightly similar C⋯Be and C⋯Mg bonds will contribute to increasing the knowledge of both the carbenes chemistry and the chemistry of CDPs. It is worth mentioning at this point that the presence of X halogen atoms leads in some of the dimers considered here to certain symptoms that indicate interactions accompanying the leading C⋯M bond. Therefore, one of the sub-goals of this article is to investigate the conditions that favor these additional weak interactions.

## 2. Results and Discussion

As mentioned in the Introduction, this article describes research on systems containing a beryllium bond, a magnesium bond or a zinc bond, where the role of Lewis acids is played by the MX${}_{2}$ molecules (where M = Be, Mg, Zn and X = H, F, Cl, Be, Me), while the role of Lewis base is played by either carbene ((NH${}_{2}$)${}_{2}$C, imidazol-2-ylidene, imidazolidin-2-ylidene, tetrahydropyrymid-2-ylidene, or cyclopropenylidene) or carbodiphosphorane (being either (PH${}_{3}$)${}_{2}$C or (NH${}_{3}$)${}_{2}$C). The monomers themselves and their dimers, in which the described bonds occur, are presented in separate subsections.

### 2.1. Investigated Systems

#### 2.1.1. Monomers

- MeX${}_{2}$

The considered MX${}_{2}$ molecules are characterized by a linear structure in which M–X bonds are formed by overlapping of the hybridized *sp* orbital of the metal atom with one of the orbitals of X. Due to lower electronegativity of the metal atom, this atom is endowed with a partial positive charge (Table 1), becoming electron-depleted and therefore a Lewis acid center.

The atomic charge values shown in Table 1 confirm the known fact that they can be very significantly dependent on the method of obtaining them in the calculations [173,174,175,176]. The atomic charges obtained by the NBO and QTAIM methods seem to be greatly exaggerated. In the context of the presented results, however, it is more important that all the methods of obtaining atomic charges used here (i.e., Hirshfeld [177,178,179], NBO [180,181], and QTAIM [182,183,184]) show that in the set of MX${}_{2}$ molecules for a given metal M the most positive charge on the M atom occurs when X = F. This is fully understandable due to the very high electronegativity of the fluorine atom. Conversely, the smallest positive charge on the M atom occurs when X = H. This result is not as expected, because, due to the positive inductive effect (+I) of the methyl group, one would expect the smallest positive atomic charge of M in MMe${}_{2}$. It is also seen that the Cl and Br atoms lead to similar atomic charges on M. Importantly, all the methods used show that the highest positive charge occurs in MgF${}_{2}$, and the lowest in ZnH${}_{2}$. If we refer to the most reliable [175,176] Hirshfeld atomic charges, then these values are 0.924 and 0.354 au, respectively. The former value suggests an extremely high polarization of the Mg-F bond, which practically becomes the Mg${}^{+}$F${}^{-}$ ionic one. A practical consequence of this finding is that, assuming electrostatic reasoning, the MgF${}_{2}$ molecule should be the best Lewis acid, and therefore it should theoretically form the strongest adducts with carbenes and CDPs.

**Figure 2:**
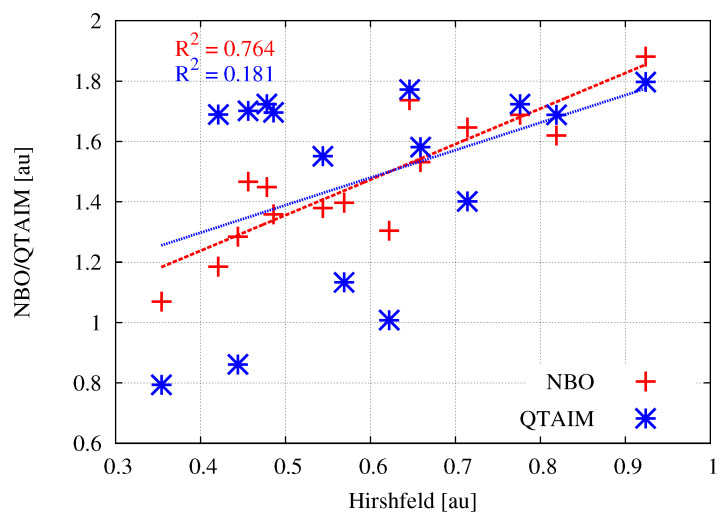
Relationships between the atomic charges of M (M = Be, Mg, Zn; see Table 1) obtained by the Hirshfeld method and either NBO or QTAIM.

With the values of the atomic charges obtained by various theoretical methods, it is interesting to see if there are clear relationships between them. Figure 2 shows the relationships between the Hirshfeld charges and their equivalents obtained by the NBO or QTAIM method.

As can be clearly seen, the linear relationships between the Hirshfeld atomic charges and those obtained by the NBO or QTAIM method are very weak. Particularly in the case of the latter method, the obtained coefficient of determination is unacceptably low. This result shows that especially the atomic charges obtained by QTAIM should not be treated as reliable. This flaw of QTAIM-based atomic charges was attributed to irregular shapes of atomic basins, which give them multipolar moments.

**Figure 3:**
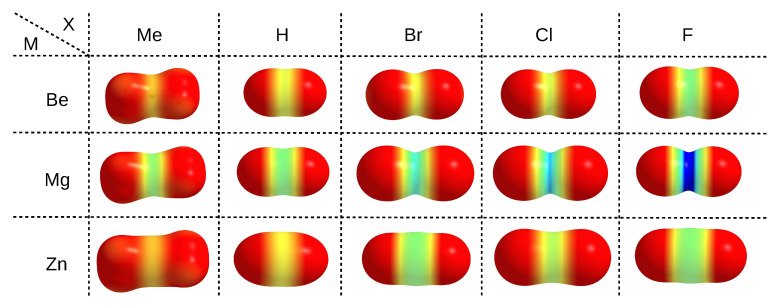
Maps of electrostatic potential projected on 0.001 au isodensity surfaces of MX${}_{2}$ (M = Be, Mg, Zn; X = Me, H, F, Cl, Br). A common value scale (in au) was used: 0.0—red, 0.05—yellow, 0.1—green, 0.15—cyan, 0.2—blue.

The electrophilic properties of a particular metal atom, which is an acidic center in the MX${}_{2}$ molecule, can be nicely illustrated by means of maps of the distribution of the molecular electrostatic potential (MESP) projected onto the electron density isosurface, as shown in Figure 3.

**Figure 4:**
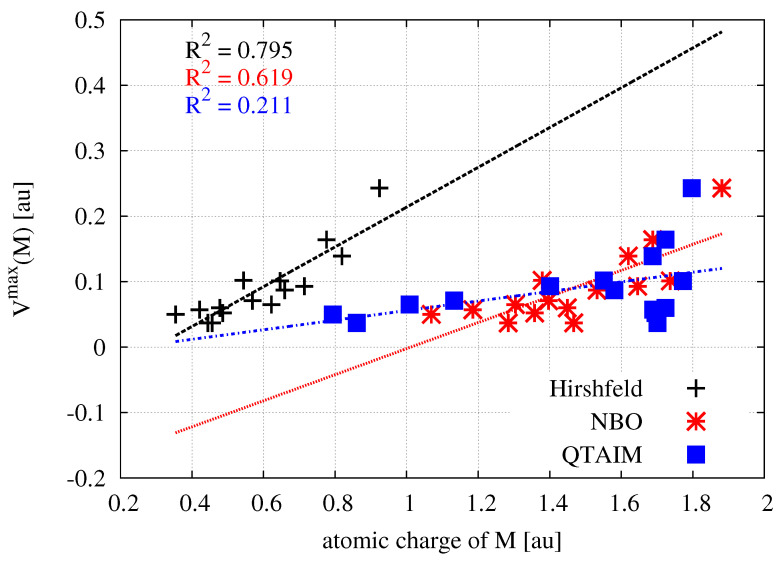
Relationships between the atomic charge of the M metal atom in MX${}_{2}$ (M = Be, Mg, Zn; X = H, F, Cl, Br, Me) and the maximum value of the electrostatic potential on the surface of this atom.

The use of same scale of the electrostatic potential values (from 0.0 au (red) to 0.2 (blue)) for all MX${}_{2}$ molecules allows one to easily capture the existing relationships. It is clearly seen that, upon going in the series Me→H→Br→Cl→F, i.e., from left to right in Figure 3, a belt of even more positive electrostatic potential develops around the central metal atom. This is of course confirmed by the corresponding values of the maximum electrostatic potential on M ($V^{\max}$(M)), which are provided in the last column of Table 1. For zinc compounds, these values (in au) increase in this series as follows: 0.037 < 0.050 < 0.065 < 0.071 < 0.093. Although the MESP maps for zinc molecules are very similar to those for beryllium, it is worth noting that in the former case, the corresponding MESP belt is wider and larger in diameter due to the larger atomic radius of Zn${}^{2+}$ (88 pm) compared to Be${}^{2+}$ (59 pm) [185]. Therefore, compared to beryllium, the zinc atom should be more accessible. The more important result, however, is that, for a given X, the belts of positive MESP are most visible when the central metal atom is magnesium. The values of $V^{\max}$(M) increase monotonically quite quickly in the order given earlier, reaching a maximum value of 0.243 au in MgF${}_{2}$ (Table 1). The fact that the $V^{\max}$(M) values increase in this order, while *q*(M) does not, suggests that $V^{\max}$(M) is perhaps the more reliable parameter describing the acidic nature of the central metal atom in MX${}_{2}$ molecules than *q*(M). Although the linear relationship between the value of $V^{\max}$(M) is not very good ($R^{2}$ = 0.795) either when *q*(M) is computed utilizing the Hirshfeld method, it is much better than in the case of NBO- and especially QTAIM-based charges (Figure 4).

- Carbenes and CDPs

Imidazol-2-ylidene, imidazolidin-2-ylidene, tetrahydropyrymid-2-ylidene, cyclopropenylidene, and (NH${}_{2}$)${}_{2}$C have been used as model representatives of carbenes. In particular, the first two carbenes are often used in organic and organometallic chemistry and represent an important starting point in the syntheses of larger carbene compounds [98,102,103]. The CDPs group is represented by (PH${}_{3}$)${}_{2}$C and its amino derivative (NH${}_{3}$)${}_{2}$C. Both are the starting molecules for more complex CDPs obtained by substituting hydrogen atoms in one or both of the -PH${}_{3}$ or -NH${}_{3}$ groups. It is worth mentioning here that the fully saturated phenyl derivative, i.e., (PPh${}_{3}$)${}_{2}$C was the first synthesized CDP [154]. Some fundamental parameters characterizing the considered carbenes and CDPs are presented in Table 2.

When analyzing the obtained values of the atomic charge on the carbon atom, one can easily notice their great diversity, even in terms of sign. In the case of carbenes, positive QTAIM atomic charges have been obtained. Additionally, this method has given (too) large variation in the negative values on the C atom in (PH${}_{3}$)${}_{2}$C and (NH${}_{3}$)${}_{2}$C (−2.261 and −0.179 au, respectively). Both of these findings strongly suggest that atomic charges of QTAIM are highly unreliable. A similar conclusion applies to the atomic charges of NBO, although the values themselves are not that large. It is worth mentioning that the value of the atomic charge on a carbon atom of −1.43 au in (PPh${}_{3}$)${}_{2}$C was used by Tonner et al. [167] as an argument supporting the bonding scheme of CDPs presented in Figure 1. However, taking into account large dependence of the atomic charge on the method used in calculations, it seems that this argument was perhaps not entirely correct. The more reliable [175,176] Hirshfeld atomic charges are negative in both carbenes and CDPs. Understandably, in the latter case they are much larger, which results from the role of the carbon atom as an acceptor in the R→C bonds (Figure 1).

**Figure 5:**
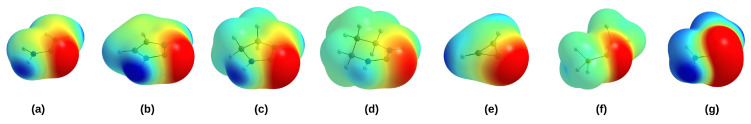
Maps of electrostatic potential projected on 0.001 au isodensity surfaces of carbenes ((**a**) (NH${}_{2}$)${}_{2}$C, (**b**) imidazol-2-ylidene, (**c**) imidazolidin-2-ylidene, (**d**) tetrahydropyrymid-2-ylidene, (**e**) cyclopropenylidene) and carbodiphosphoranes ((**f**) (PH${}_{3}$)${}_{2}$C, (**g**) (NH${}_{3}$)${}_{2}$C). A common value scale (in au) was used: −0.06—red, −0.03—yellow, 0.00—green, 0.03—cyan, 0.06—blue.

Further valuable information on the nucleophilic abilities of singlet carbenes and CDPs can be obtained from the values of the minimum electrostatic potential on C (the penultimate column in Table 2) and the distribution of this potential around this atom (see Figure 5).

The electrostatic potential maps clearly show the negative potential area around the C(2) atom in the carbenes or the C(0) atom in the CDPs. On the other hand, strong positive potential concerns mainly hydrogen atoms in strongly polar N-H bonds. While the characteristics of the negative potential distribution around the carbon atom are similar in carbenes (which is in line with the rather similar values of $V^{\min}$(C); Table 2), there is a clear difference between (PH${}_{3}$)${}_{2}$C and (NH${}_{3}$)${}_{2}$C. Specifically, in the latter case, this area in much clearer and much more spread around the carbon atom, which better emphasizes the great nucleophilic properties of this molecule. Both of these molecules also differ considerably in the value of $V^{\min}$(C) (−0.067 and −0.109 au, respectively).

**Figure 6:**
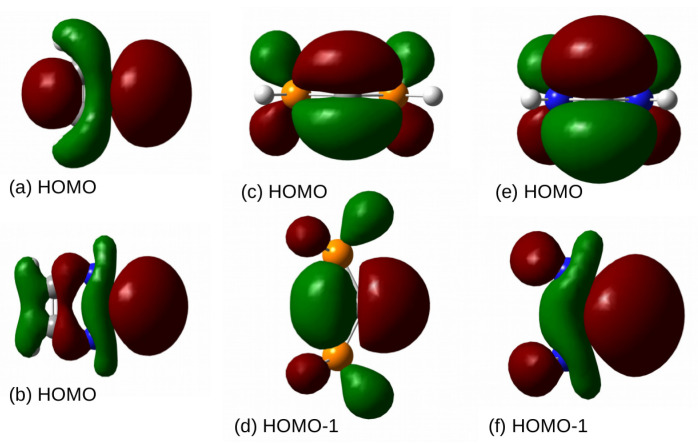
Molecular orbitals of carbenes and CDPs: (**a**) HOMO of cyclopropenylidene, (**b**) HOMO of imidazol-2-ylidene, (**c**) HOMO of (PH${}_{3}$)${}_{2}$C, (**d**) HOMO-1 of (PH${}_{3}$)${}_{2}$C, (**e**) HOMO of (NH${}_{3}$)${}_{2}$C, (**f**) HOMO-1 of (NH${}_{3}$)${}_{2}$C.

Further information on the reactivity of molecules can be obtained from the Frontier Molecular Orbital theory [186,187], which has found its mathematical support in the Klopman–Salem Equation [188,189]. According to it, the electron-donating properties of the molecule can be characterized by the energy of HOMO. These energies for carbenes and CDPs are shown in the last column of Table 2. By far the least negative value of the HOMO energy obtained for (NH${}_{3}$)${}_{2}$C (−4.35 eV) confirms that this molecule should undoubtedly be the most reactive, willingly acting as a Lewis base. It should be noted, however, that the HOMO energy, like the LUMO energy, which is also often used in the Frontier Molecular Orbital theory, is a global quantity, i.e., resulting from the electronic structure of the entire molecule, and therefore it does not necessarily correctly assess the nucleophilic and electrophilic properties of a molecule, which are most often strongly local. Moreover, these energies do not necessarily correlate well with the parameters characterizing the dimer strength. For example, as shown by Martín-Sómer et al. [24], LUMO energies correlate well with interaction energies (of some beryllium bonds) only when they are computed for acceptor molecules in their dimer geometries. For this reason, LUMO energy values for the fully optimized MX${}_{2}$ molecules were not exposed in Table 2. Moreover, in the case of MX${}_{2}$ molecules, the LUMO energy strongly depends on the X-M-X angle ($\alpha_{\mathrm{XMX}}$), decreasing considerably with increasing deviation from the linearity of the molecule. In this way, Martín-Sómer et al. [24] explained the large non-linearity of the BeH${}_{2-n}$X${}_{n}$ (X = F, Cl, Br; n≤2) molecules in their dimers with ammonia. Therefore, it seems that there is nothing to prevent the same cause of MX${}_{2}$ bending also working for other Lewis bases, such as the carbenes and CDPs considered here. It is also worth mentioning that the electron lone pair, which in carbenes is HOMO (quite strongly delocalized), in the case of CDPs, i.e., (PH${}_{3}$)${}_{2}$C and (NH${}_{3}$)${}_{2}$C, becomes HOMO-1, while HOMO is the electron lone pair perpendicular to the plane of the molecule (Figure 6).

#### 2.1.2. Dimers

**Figure 7:**
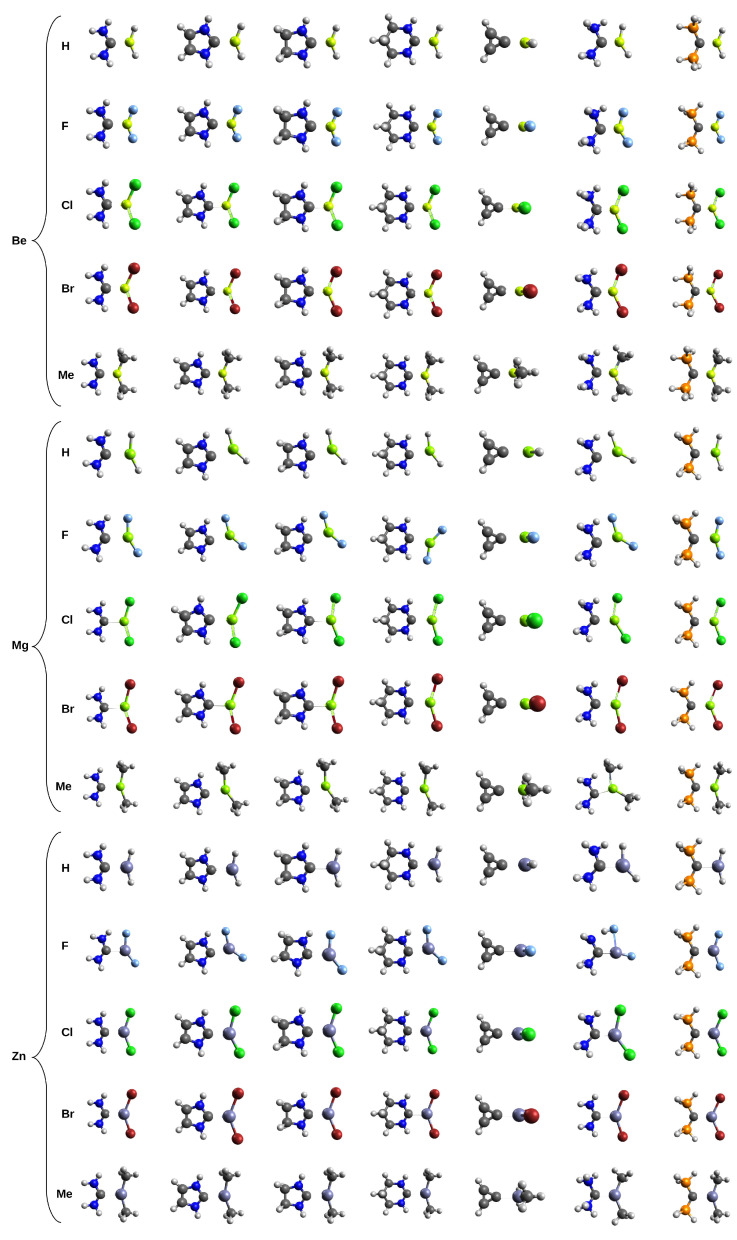
Geometries of the fully optimized dimers.

The previous subsection has shown that in MX${}_{2}$ (M = Be, Mg, Zn; X = H, F, Cl, Br, Me) molecules, the metal atom is a relatively strong acid center, while the C(2) atoms in the carbenes and C(0) in the CDPs are strong basic regions. Moreover, these atoms are the only such regions in these molecules (see Figure 3 and Figure 5). Due to this alignment in electronic properties, it should be expected that the MX${}_{2}$ molecules quite easily form a M⋯C bond to the C(2) carbon in carbenes or C(0) in CDPs. If so, it should lead to a particular type of beryllium, magnesium, or zinc (spodium) bond. As mentioned in the Introduction, the main purpose of this article is to describe these interactions. Nevertheless, the electrostatic potential distributions for MX${}_{2}$ (Figure 3) and carbenes and CDPs (Figure 5) suggest that other interactions accompanying the leading M⋯C interaction may also be possible. In particular, some symptoms of the presence of a hydrogen bond of the N-H⋯X type (where X is a halogen atom, especially F) are to be expected. The geometries of the fully optimized dimers are shown in Figure 7. It is convenient to describe the characteristics of the systems containing carbenes and CDPs separately.

- Carbene dimers

The basic parameters characterizing the investigated carbene-containing dimers are shown in Table 3.

Due to the simple structure of the cyclopropenylidene molecule, dimers containing this carbene will be discussed first. It should be noted that the plane of the slightly bent MX${}_{2}$ molecule is oriented perpendicular to the plane of the cyclopropenylidene ring (Figure 7). For this reason, the interaction between MX${}_{2}$ and cyclopropenylidene is free from any other significant interactions than C⋯M. Although the earlier analysis of the values of atomic charges and electrostatic potentials on M and C suggests that the strongest C⋯M interaction should be present in the case of MgF${}_{2}$ and the weakest in the case of ZnH${}_{2}$, this is not in line with the values of the distance C⋯M ($d_{C\cdots M}$). Rather, these distances result from the radius of the metal atom, so in the case of beryllium, $d_{C\cdots M}$ is less than ca. 1.83 Å, while in the case of Mg and Zn, this distance is over 2 Å. The penultimate column in Table 3 shows that cyclopropenylidene⋯MX${}_{2}$ dimers are strongest (32–35 kcal/mol) when M is either Be or Mg and X is a halogen, especially Cl or Br. The lowest dissociation energy (10.3 kcal/mol) has been obtained in the case of ZnMe${}_{2}$. The weakest C⋯M in the presence of methyl groups has also been obtained in the case of M = Be or Mg and is in line with the weak +I character of the methyl group. Due to the C${}_{2V}$ symmetry, the following relations hold: $\Delta d_{\mathrm{MX}1}$ = $\Delta d_{\mathrm{MX}2}$ = $\Delta d_{\mathrm{MX}}^{\mathrm{av}}$ and $\alpha_{\mathrm{CMX}1}$ = $\alpha_{\mathrm{CMX}2}$. The greatest elongation of the MX bond (0.093 Å) occurs in BeBr${}_{2}$. Along with a similar BeCl${}_{2}$, in this molecule, there is also the greatest deviation from linearity ($\alpha_{\mathrm{XMX}}$ amounts to ca. 134${}^{\circ }$). Thus, the geometric characteristics of the MX${}_{2}$ molecule itself and the obtained $D_{0}$ values suggest that in the cyclopropenylidene⋯MX${}_{2}$ dimers, the interaction should be strongest for BeBr${}_{2}$ and BeCl${}_{2}$. It is interesting to see if similar finding also apply to dimers involving the other carbenes.

As Figure 7 shows, the MX${}_{2}$ molecule lies in the same plane as the backbone atoms of the carbene molecule. This arrangement is also characteristic for dimers involving CDPs, (PH${}_{3}$)${}_{2}$C and (NH${}_{3}$)${}_{2}$C. In at least some cases, the planar geometry of the dimer can be explained by additional beneficial interactions (as will be discussed). As was the case with cyclopropenylidene, the intermolecular distance C⋯M is much shorter for beryllium (ca. 1.76–1.85 Å) than for either magnesium (ca. 2.17–2.28 Å) or zinc (2.03–2.20 Å). However, this does not mean stronger C⋯M interactions. The calculated dissociation energy values clearly show (Table 3) that, as was the case for cyclopropenylidene, the strongest intermolecular C⋯M interaction occurs for BeBr${}_{2}$ and BeCl${}_{2}$. Although the bond strength of the former is ca. 47–48 kcal/mol, it reaches up to 53 kcal/mol when BeBr${}_{2}$ interacts with tetrahydropyrymid-2-ylidene. On the other hand, similar to cyclopropenylidene, the C⋯M interaction is the weakest (but clearly stronger than that of cyclopropenylidene) when the MX${}_{2}$ molecule is ZnMe${}_{2}$. Consequently, in the dimers considered here, the dissociation energies of C(2)⋯M have a wide range from 10 to 53 kcal/mol. This result is in full accord with the recent generalization given by Alkorta and Legon that beryllium and magnesium bonds (the current results show also include the zinc bonds) are generally much stronger than hydrogen bonds, halogen bonds, etc. [29].

**Figure 8:**
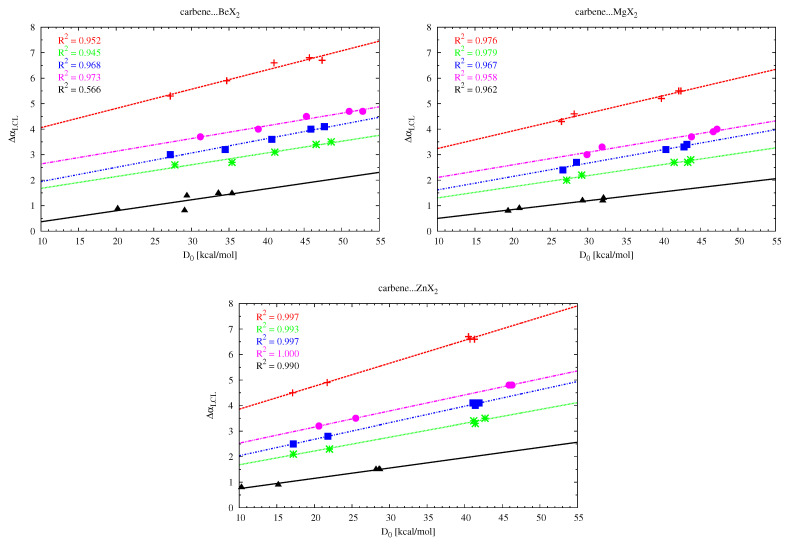
Relationships between the change of the LCL opening angle ($\Delta \alpha_{\mathrm{LCL}}$) and the dissociation energy ($D_{0}$) of the L${}_{2}$C⋯MX${}_{2}$ dimers, where L${}_{2}$C = (NH${}_{2}$)${}_{2}$C (red), imidazol-2-ylidene (green), imidazolidin-2-ylidene (blue), tetrahydropyrymid-2-ylidene (magenta), cyclopropenylidene (black).

The LCL angle change ($\Delta \alpha_{\mathrm{LCL}}$) values show that the interaction between MX${}_{2}$ and the carbene molecule leads to the opening of the latter molecule, with the effect being the greatest for (NH${}_{2}$)${}_{2}$C (e.g., $\Delta \alpha_{\mathrm{LCL}}$ = 6.8${}^{\circ }$ for (NH${}_{2}$)${}_{2}$C⋯BeCl${}_{2}$). This shows that the $\alpha_{\mathrm{NCN}}$ angle in (NH${}_{2}$)${}_{2}$C is more flexible than in cyclic and therefore more rigid imidazol-2-ylidene, imidazolidin-2-ylidene and tetrahydropyrymid-2-ylidene ($\Delta \alpha_{\mathrm{CCC}}$ in cyclopropenylidene is negligible). Although in general the $\alpha_{\mathrm{NCN}}$ angle-opening effect in the carbene molecule does not seem to be dependent on the strength of the interaction with MX${}_{2}$, such a relationship can be found when comparing systems with similar skeleton stiffness. Therefore, in the group of the aforementioned cyclic carbenes, the strongest effect occurs in tetrahydropyrymid-2-ylidene (4.8${}^{\circ }$). Excellent linear relationships have been found (see Figure 8) between the change of the opening angle $\alpha_{\mathrm{LCL}}$ and the dissociation energy of the carbene⋯MX${}_{2}$ dimer as long as the carbenes and the MX${}_{2}$ molecules are treated separately. Note that the greater sensitivity of the opening angle in the case of the (NH${}_{2}$)${}_{2}$C carbene is evident here by slightly larger slopes of the corresponding (red) lines. Moreover, the slopes of the linear fits for cyclic carbenes are similar to each other.

**Figure 9:**
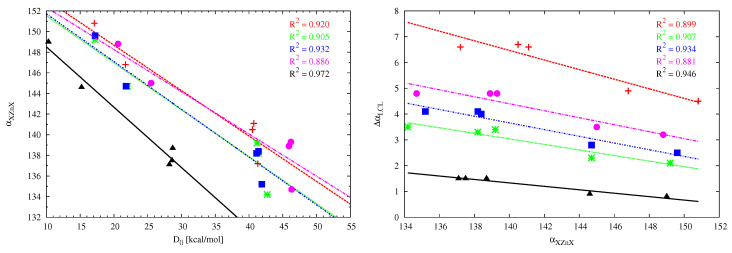
Relationships between either the XZnX angle ($\alpha_{\mathrm{XZnX}}$) and the dissociation energy ($D_{0}$) (left) or the change of the LCL angle ($\Delta \alpha_{\mathrm{LCL}}$) and $\alpha_{\mathrm{XZnX}}$ (right) for the L${}_{2}$C⋯ZnX${}_{2}$ dimers (where L${}_{2}$C = (NH${}_{2}$)${}_{2}$C (red), imidazol-2-ylidene (green), imidazolidin-2-ylidene (blue), tetrahydropyrymid-2-ylidene (magenta), cyclopropenylidene (black)).

A characteristic effect that occurs during an interaction of the initially linear MX${}_{2}$ molecule with a strong Lewis base is its significant bend [23]. For example, Martín-Sómer et al. have reported XCX angles ($\alpha_{\mathrm{XMX}}$) of 134${}^{\circ }$–139${}^{\circ }$, (B3LYP/6-311+G(3df,2p)) for dimers of X-substituted (X = F, Cl, Br) BeX${}_{2}$ derivatives with ammonia [24]. This bending effect is much less (138${}^{\circ }$–149${}^{\circ }$,) in BeX${}_{2}$ (X = H, F, Cl) dimers with ethylene or acetylene, being much weaker Lewis bases interacting via π bonds [26]. The high sensitivity of the $\alpha_{\mathrm{XMX}}$ angle makes it particularly interesting to trace its values in the considered dimers. Due to the large number of the studied set of systems and their diversity (different acid centers M, different X substituents, different carbenes), a fairly wide range of $\alpha_{\mathrm{XMX}}$ variability has been obtained, from 131 to 151${}^{\circ }$, i.e., as much as 20${}^{\circ }$. The bending effect is greatest for BeCl${}_{2}$ and BeBr${}_{2}$ and the smallest for ZnMe${}_{2}$. The linear correlation between the XMX angle and the dissociation energy is acceptable for ZnX${}_{2}$ (Figure 9, left) and the dimers of either imidazol-2-ylidene ($R^{2}$ = 0.942) or imidazolidin-2-ylidene ($R^{2}$ = 0.922) with BeX${}_{2}$ (not shown). The fitting line for cyclopropenylidene has slightly different slope than the other four cases, which may result from different (perpendicular) orientation of the interacting molecules (Figure 7). The weak linear correlation for the remaining cases of carbene⋯MX${}_{2}$ (M = Be, Mg) dimers may, at least partly, result from the presence of additional interactions is some of the considered dimers, which should have some influence on the angle XMX. In the case of the dimers involving ZnX${}_{2}$, as a consequence of good linear relationships between $\Delta \alpha_{\mathrm{LCL}}$ and $D_{0}$ (Figure 8) and $\alpha_{\mathrm{XZnX}}$ and $D_{0}$ (Figure 9, left), one also observes good linear relationships between $\Delta \alpha_{\mathrm{LCL}}$ and $\alpha_{\mathrm{XZnZ}}$ (Figure 9, right).

Another effect observed during the formation of the carbene⋯MX${}_{2}$ dimers is a significant elongation of both MX bonds. It should be clearly underlined here that, in general, both MX bond elongations are not necessarily of equal magnitude, so it is not necessarily true that $\Delta d_{\mathrm{MX}1}$ = $\Delta d_{\mathrm{MX}2}$ = $\Delta d_{\mathrm{MX}}^{\mathrm{av}}$ (Table 3). These unsymmetrical elongations of MX result from the presence of certain accompanying interactions in some of the dimers studied here. Such cases are also clearly visible from different values of CMX1 and CMX2 angles ($\alpha_{\mathrm{CMX}1}$ and $\alpha_{\mathrm{CMX}2}$, respectively) in Table 3. In such cases, the smaller of these angles ($\alpha_{\mathrm{CMX}1}$) takes a value roughly about the right angle.

As already mentioned, any significant additional interactions are impossible in cyclopropenylidene dimers. In this case, the effect of MX bond elongation is therefore symmetrical, which allows for straigtforward analysis of the obtained relationships. The greatest elongation of the MX bonds is for X = Cl or Br, but only when the M atom is either beryllium or zinc (up to 0.093 Å for BeBr${}_{2}$). Hence, the elongation effect is not entirely consistent with the strength of C⋯M if measured by $D_{0}$. On the contrary, the smallest elongation of the MX bond occurs in MgMe${}_{2}$ (0.030 Å) and MgF${}_{2}$ (0.031 Å). Although the relatively small magnitude of the effect in the former case can be explained by a relatively weak interaction (the largest distance C⋯M amounting to 2.288 Å and the smallest bending of 149.1${}^{\circ }$), BeH${}_{2}$ is also characterized by a small MX bond elongation (0.039 Å), and this molecule forms the shortest contact with cyclopropenylidene, amounting to only 1.743 Å. For the latter molecule, i.e., MgF${}_{2}$, the small effect of the MgF bond elongation can most likely be explained by a high polarity of the bond and therefore its considerable resistance to changes. It seems that the magnitude of the MX bond elongation does not clearly depend on $d_{C\cdots M}$ or the interaction strength measured by $D_{0}$.

As mentioned earlier, the asymmetry of the MX elongation effects in case of many dimers involving the remaining carbenes makes the analysis much more difficult, but mean value ($\Delta d_{\mathrm{MX}}^{\mathrm{av}}$) provides some information. Regardless of carbene, this value for BeBr${}_{2}$ is always greater than 0.106 Å and reaches a maximum value of 0.111 Å for tetrahydropyrymid-2-ylidene, thus confirming that presumably the C⋯M interaction is the strongest in the tetrahydropyrymid-2-ylidene⋯BeBr${}_{2}$ dimer. The occurrence of the minimum values of $\Delta d_{\mathrm{MX}}^{\mathrm{av}}$ appears to be more irregular. Although BeH${}_{2}$ is generally characterized by low values (ca. 0.048 Å), the lowest values (ca. 0.040 Å) are nevertheless found for MgMe${}_{2}$ interacting with either imidazol-2-ylidene or imidazolidin-2-ylidene.

**Figure 10:**
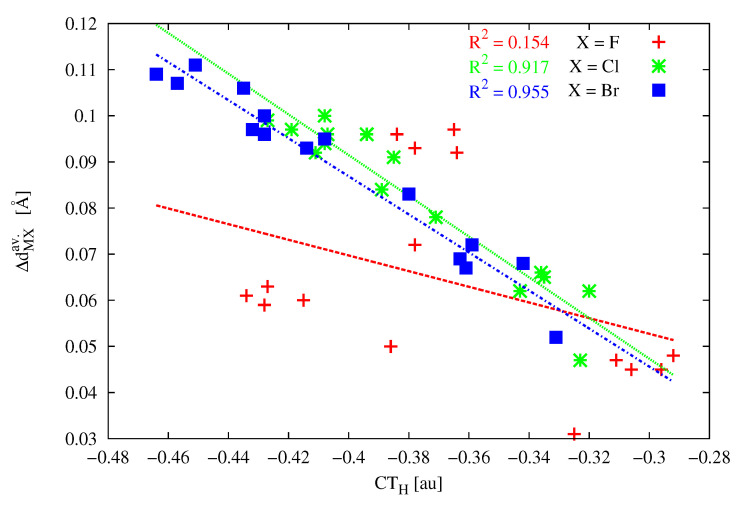
Relationship between charge transfer (CT${}_{H}$) and the averaged elongation of the MX bond ($\Delta d_{\mathrm{MX}}^{\mathrm{av}.}$).

The last column in Table 3 shows values of charge transfer calculated by means of the most reliable [175,176] Hirshfeld atomic charges (CT${}_{H}$). First, it should be noted that the obtained values are negative, which means that the formation of the carbene⋯MX${}_{2}$ dimer leads to an increase in the total charge on the MX${}_{2}$ molecule. Secondly, the obtained values are very large. Suffice it to mention that the corresponding charge transfer values obtained (on the same level of theory) for dimers HOH⋯OH${}_{2}$ and HOH⋯NH${}_{3}$ are −0.098 and −0.122 au, respectively. Thus, even the weakest charge transfers obtained for the investigated dimers are over two times greater (e.g., −0.270 au for cyclopropenylidene⋯ZnMe${}_{2}$) and even reach almost four times higher values in some dimers with BeBr${}_{2}$ (e.g., CT${}_{H}$ amounts to ca. −0.46 au for imidazol-2-ylidene, imidazolidin-2-ylidene, and tetrahydropyrymid-2-ylidene). Undoubtedly, therefore, the carbene⋯MX${}_{2}$ (M = Be, Mg, Zn) dimers considered here are characterized by a significant charge transfer, which is particularly high in the presence of highly polarizable halogen atoms in MX${}_{2}$, especially Br. This finding is also manifested by very good ($R^{2}$ = 0.955) linear correlation between CT${}_{H}$ and $\Delta d_{\mathrm{MX}}^{\mathrm{av}.}$ when X = Br and only slightly worse for X = Cl ($R^{2}$ = 0.917), while this correlation is very weak ($R^{2}$ = 0.154) for much less polarizable fluorine (Figure 10).

**Figure 11:**
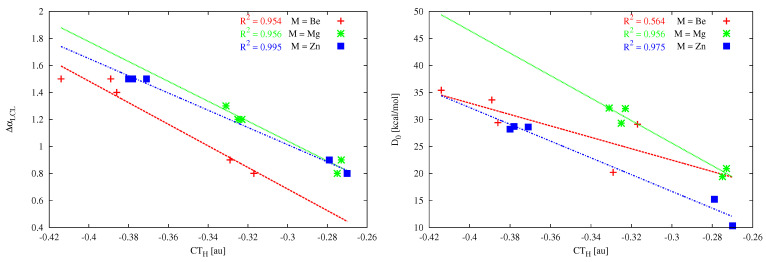
Relationships between charge transfer (CT${}_{H}$) and either the change in the LCL angle ($\Delta \alpha_{\mathrm{LCL}}$) or the dissociation energy ($D_{0}$) obtained for the cyclopropenylidene⋯MX${}_{2}$ (M = Be, Mg, Zn) dimers.

For the cyclopropenylidene⋯MX${}_{2}$ dimers, there are also very good linear correlations between CT${}_{H}$ and either $\Delta \alpha_{\mathrm{LCL}}$ or D${}_{0}$ (in the latter relationship, except in the case of M = Be) if only systems with different M atoms are treated separately (Figure 11).

Unfortunately, similar relationships are generally much worse for other carbenes, which can be explained by the presence of additional intermolecular interactions in some of them, which to some extent affects the obtained values of the analyzed parameters.

- CDPs dimers

The fundamental data characterizing CDP⋯MX${}_{2}$ dimers are included in Table 4.

Its penultimate column shows that the C(0)⋯M interactions in the dimers formed by (PH${}_{3}$)${}_{2}$C are comparable in strength to the C(2)⋯M bonds formed by the investigated carbenes, whereas those formed by (NH${}_{3}$)${}_{2}$C are much stronger. Again, the maximum value is found for BeBr${}_{2}$, reaching 84 kcal/mol, a value comparable to the energy of weaker covalent bonds [190]. The other dimers with high values of $D_{0}$ are (NH${}_{3}$)${}_{2}$C⋯BeCl${}_{2}$ and (NH${}_{3}$)${}_{2}$C⋯ZnF${}_{2}$ (ca. 79 kcal/mol). It is worth recalling here the theoretical research by Jabłoński and Palusiak [108] on the ability of carbenes and CDPs to form hydrogen bonds. The results of those studies have shown that for the same Lewis acid (e.g., HCCH), the hydrogen bond to (NH${}_{3}$)${}_{2}$C is much stronger than to (PH${}_{3}$)${}_{2}$C (the MP2/aug-cc-pVTZ-based BSSE-corrected interaction energies amount to −9.16 and −5.31 kcal/mol, respectively, [108]), which further confirms the greater basicity of the former molecule. Although the (NH${}_{3}$)${}_{2}$C⋯BeBr${}_{2}$ and (NH${}_{3}$)${}_{2}$C⋯BeCl${}_{2}$ dimers are characterized by short C⋯Be distances (1.643 and 1.661 Å, respectively), the short C⋯Be distance (1.655 Å) is also present in the (NH${}_{3}$)${}_{2}$C⋯BeH${}_{2}$ dimer with much weaker interaction (64 kcal/mol).

Very high bond strength of C(0)⋯M in the (NH${}_{3}$)${}_{2}$C⋯MX${}_{2}$ dimers is in line with high values of charge transfer, which can even reach -0.610 au in the (NH${}_{3}$)${}_{2}$C⋯BeBr${}_{2}$ dimer. This value is more than six times greater than that of the water dimer and exactly five times greater than that of the water-ammonia dimer. A curiosity is the relatively low CT${}_{H}$ value (−0.287 au) obtained for the (NH${}_{3}$)${}_{2}$C⋯ZnF${}_{2}$ dimer with a simultaneous very high dissociation energy (78.8 kcal/mol). In the next subsection, however, it will be shown that this dimer is characterized by a highly advanced proton transfer from N to F, which results in the formation of the N⋯H-F hydrogen bond. The formation of the H-F bond requires some removal of the electron charge from the fluorine atom.

An interesting result is that, as in the case of carbenes (Table 3), the interaction between MX${}_{2}$ and (NH${}_{3}$)${}_{2}$C causes a significant opening of the $\alpha_{\mathrm{NCN}}$ angle, whereas in the case of (PH${}_{3}$)${}_{2}$C, the change in the $\alpha_{\mathrm{PNP}}$ angle is much smaller and may have different sign, most often being negative. This finding clearly differentiates the nitrogen atom from the phosphorus atom.

**Figure 12:**
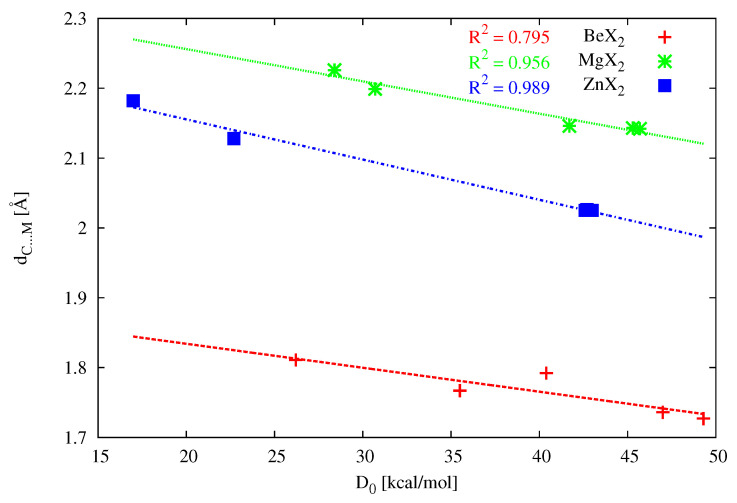
Relationships between $d_{C\cdots M}$ and $D_{0}$ obtained for (PH${}_{3}$)${}_{2}$C⋯MX${}_{2}$ (X = Be, Mg, Zn) dimers.

The comparison of the values of $\Delta d_{\mathrm{MX}1}$ and $\Delta d_{\mathrm{MX}2}$ as well as $\alpha_{\mathrm{CMX}1}$ and $\alpha_{\mathrm{CMX}2}$ shows a clear difference between the dimers with (PH${}_{3}$)${}_{2}$C and the dimers with (NH${}_{3}$)${}_{2}$C. Specifically, the former of them are characterized by the equality of both quantities, which indicates symmetry of these dimers with respect to the axis passing through the C and M atoms (see also Figure 7). In the latter case, however, this symmetry is clearly broken in most of the dimers, which results from the presence of other interactions accompanying the leading contact C⋯M. As a result, the search for linear correlations between the parameters from Table 4 for systems with (NH${}_{3}$)${}_{2}$C is pointless, while the search for such correlations for systems with (PH${}_{3}$)${}_{2}$C seems to be justified. Indeed, some reasonable linear correlations have been found, such as, for example, between $d_{C\cdots M}$ and $D_{0}$ (see Figure 12) when the metal atom is either Mg ($R^{2}$ = 0.956) or especially Zn ($R^{2}$ = 0.989). On the other hand, when the acidic metal center is beryllium, the linear correlation clearly deteriorates ($R^{2}$ = 0.795). At least in part, this may be due to the much shorter Be-X bond compared to the Mg-X or Zn-X bonds, and thus stronger, although still rather weak, intermolecular interactions of the type -(PH${}_{3}$)⋯X. Moreover, quite good linear relationships between CT${}_{H}$ and $d_{C\cdots M}$ ($R^{2}$ = 0.901), $\Delta d_{\mathrm{MX}}^{\mathrm{av}.}$ ($R^{2}$ = 0.911) or $D_{0}$ ($R^{2}$ = 0.930) have been found for the analyzed (PH${}_{3}$)${}_{2}$C⋯ZnX${}_{2}$ dimers.

### 2.2. Other Accompanying Interactions

**Figure 13:**
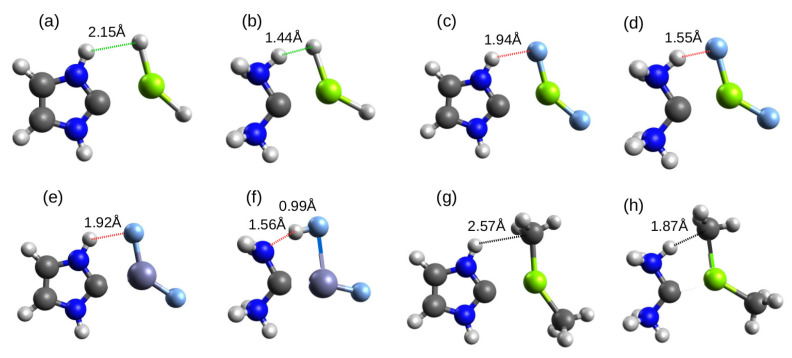
Structures of selected dimers containing certain intermolecular interactions accompanying the C⋯M bond: (**a**) imidazol-2-ylidene⋯MgH${}_{2}$, (**b**) (NH${}_{3}$)${}_{2}$C⋯MgH${}_{2}$, (**c**) imidazol-2-ylidene⋯MgF${}_{2}$, (**d**) (NH${}_{3}$)${}_{2}$C⋯MgF${}_{2}$, (**e**) imidazol-2-ylidene⋯ZnF${}_{2}$, (**f**) (NH${}_{3}$)${}_{2}$C⋯ZnF${}_{2}$, (**g**) imidazol-2-ylidene⋯MgMe${}_{2}$, (**h**) (NH${}_{3}$)${}_{2}$C⋯MgMe${}_{2}$.

As mentioned earlier, some of the obtained structures of the studied dimers suggest presence of an additional intermolecular interaction that accompanies the described beryllium, magnesium, or zinc bonds to the carbene or carbodiphosphorane carbon atom. As these interactions are of various types, it is worth showing some of the more interesting examples obtained. Most of these dimers contain imidazol-2-ylidene or (NH${}_{3}$)${}_{2}$C; therefore, the examples shown for the reader’s convenience in Figure 13 refer to these molecules.

The fact that it is imidazol-2-ylidene and (NH${}_{3}$)${}_{2}$C molecules that willingly form different interactions with MX${}_{2}$ than just C⋯M contact could have been expected from the electrostatic potential distribution map shown in Figure 5. From these maps, it is clear that both of these molecules have most acidic regions associated with the highly polar N-H bonds. It is therefore to be expected that these molecules will readily form a dihydrogen or hydrogen bond when the appropriate opportunity arises. Figure 13 shows that this is indeed the case. In cases (a) and (b), in addition to a magnesium bond to the carbon atom, there is also a dihydrogen bond. In the latter case it is very short, because the H⋯H distance is only 1.44 Å. It should be emphasized that it is not common for the dihydrogen bond between neutral molecules to be so short [191]. Of course, an acidic hydrogen atom from the N-H bond easily forms a hydrogen bond as well, as long as its acceptor is a strongly electronegative atom like fluorine, e.g., in MgF${}_{2}$ (subfigures (c) and (d)). Again, this bond is clearly shorter (1.55 Å vs. 1.94 Å) when the N-H donor bond belongs to (NH${}_{3}$)${}_{2}$C. In the case of imidazol-2-ylidene⋯ZnF${}_{2}$ (e), the length of the N-H⋯F hydrogen bond is 1.92 Å, therefore similar to imidazol-2-ylidene⋯MgF${}_{2}$ (c). However, in (NH${}_{3}$)${}_{2}$C⋯ZnF${}_{2}$ (f), the interaction between H and F is so strong that there is a highly advanced proton transfer to F, so that the distance H⋯F becomes much shorter (0.99 Å) than N⋯H (1.56 Å). Therefore, in this case, it is more logical to speak of a hydrogen bond of the F-H⋯N type. On the other hand, the dimers marked in Figure 13 as (g) and (h) are examples of systems with rather non-standard hydrogen bonds of the N-H⋯C type. In this pair, again, the interaction is much shorter for (NH${}_{3}$)${}_{2}$C (1.87 Å) than for imidazol-2-ylidene (2.57 Å). The discussed examples are good illustrations of the coexistence of two formally completely different intermolecular interactions. Obviously, such an occurrence makes it much more difficult to extract the characteristic features for just one of them—in this case, the magnesium or zinc (spodium) bond.

### 2.3. QTAIM- and NCI-Based Characteristics

The characteristics of the studied dimers can be further investigated using the QTAIM [182,183,184] and NCI [192,193] theoretical methods. In particular, the former one is one of the most frequently used in studies of various intermolecular interactions. On the other hand, the latter of these methods is much less frequently used, and, to my knowledge, has not yet been utilized in the study of beryllium, magnesium, or zinc bonds.

#### 2.3.1. QTAIM

As QTAIM has already been used previously for describing beryllium and magnesium bonds in some simple dimers [23,24,25,26,27], the main focus in this subsection is on the characteristics of the zinc bond and its possible differences from beryllium and magnesium bonds. For this purpose, QTAIM calculations were performed (ωB97X-D/6-311++G(2df,2pd)) for the following representative dimers: cyclopropenylidene⋯MX${}_{2}$ (X = H, Br), imidazol-2-ylidene⋯MBr${}_{2}$, (PH${}_{3}$)${}_{2}$C⋯MBr${}_{2}$, and (NH${}_{3}$)${}_{2}$C⋯MBr${}_{2}$, where M = Be, Mg, Zn. These dimers were also chosen because they are examples of the dimers in which the previously described accompanying interactions either do not exist or do not have so significant influence on the C⋯M bond. Values of the most important quantities obtained by means of QTAIM are shown in Table 5.

It is worth noting at the beginning that in terms of the obtained electron density ($\rho_{C\cdots M}$) values or the total electronic energy density ($H_{C\cdots M}$) calculated at the critical point of the C⋯M bond, the zinc bond does not differ much from the beryllium bond, whereas the corresponding values determined at the critical point of the magnesium bond are clearly different. For example, for the cyclopropenylidene⋯MH${}_{2}$ dimer, the following values of $\rho_{C\cdots M}$ and $H_{C\cdots M}$, respectively, for M = Zn, Be, and Mg have been obtained (in au): 0.074 ≈ 0.076 ≉ 0.033 and −0.023 ≈−0.022 ≉ 0.003. Similarly, the corresponding pairs of triples of values for imidazol-2-ylidene⋯MBr${}_{2}$ are: 0.093 ≈ 0.082 ≉ 0.046 and −0.032 ≈−0.031 ≉ 0.000 and for (PH${}_{3}$)${}_{2}$C⋯MBr${}_{2}$: 0.089 ≈ 0.085 ≉ 0.045 and −0.032 ≈−0.036 ≉−0.001. The similarity in terms of $\rho_{C\cdots M}$ and $H_{C\cdots M}$ of the zinc bond to the beryllium bond found here is an important result because, unlike the beryllium bonds [23,24,25,26,27,28,29,30], the former are studied only sporadically [91,93].

**Figure 14:**
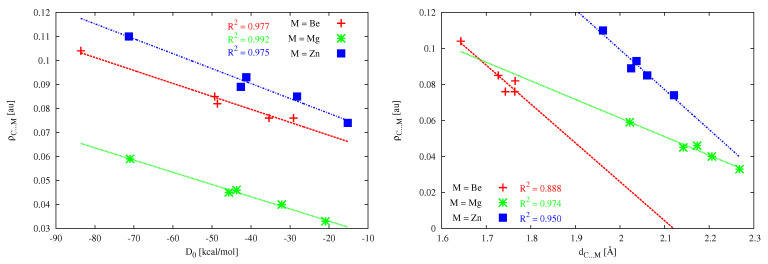
The relationships between the electron density at the bond critical point of the C⋯M interaction ($\rho_{C\cdots M}$) and either the dimer dissociation energy (**left**) or the C⋯M distance (**right**) determined for the dimers in Table 5.

For various types of interactions, the value of the electron density determined at the bond critical point of a given interaction (bond) is often treated as a measure of the strength of this interaction [182]. If so, then the zinc bonds should have a similar strength to the beryllium bonds (or even they should be slightly stronger than them), whereas the magnesium bonds should be much weaker. Comparison of the corresponding values of $\rho_{C\cdots M}$ for the dimers with the same MBr${}_{2}$ molecule suggests that cyclopropenylidene dimers should be the weakest, whereas (NH${}_{3}$)${}_{2}$C dimers should be by far the strongest. The dimers with imidazol-2-ylidene or (PH${}_{3}$)${}_{2}$C should have similar strength and intermediate between cyclopropenylidene and (NH${}_{3}$)${}_{2}$C. For example, in the case of ZnBr${}_{2}$, the $\rho_{C\cdots M}$ values (in au) for cyclopropenylidene, (PH${}_{3}$)${}_{2}$C, imidazol-2-ylidene, and (NH${}_{3}$)${}_{2}$C are, respectively, 0.085 ⪅ 0.089 ⪅ 0.093 ≪ 0.110. It is interesting to see if there is a good linear correlation between the calculated values of dissociation energies ($D_{0}$) and the $\rho_{C\cdots M}$ values shown in Table 5. The corresponding relationships are shown in Figure 14 (left). They illustrate that, indeed, the relationships between $\rho_{C\cdots M}$ and $D_{0}$ are very good ($R^{2}>0.975$), as long as the dimers with different M are treated separately.

It should also be interesting to check the quality of the linear relationship between the values of $\rho_{C\cdots M}$ and $d_{C\cdots M}$. This relationship for the different M atoms is also shown in Figure 14 (right). For Mg and Zn, the coefficients of determination are very good (0.974 and 0.950, respectively), whereas the linear correlation is clearly worse for Be (0.888). This may result from much shorter C⋯M distances, and thus stronger intermolecular interactions. It is worth noting here similar slopes of the fitting lines for Be and Zn, which again supports the previously shown similarity of the beryllium and zinc bonds in the systems considered. On the other hand, the slope of the appropriate linear fit for Mg is much smaller, thus showing a much weaker relationship between the C⋯M distance and $\rho_{C\cdots M}$ in the analyzed group of dimers.

The positive values (Table 5) of the Laplacian of the electron density at the bond critical point of C⋯M ($\nabla^{2}\rho_{C\cdots M}$) show that this interaction is of closed-shell type [182]. However, all the complexes with Zn or Be taken into account in Table 5 feature a significantly negative value of the total electronic energy density at the bond critical point of C⋯M (i.e., $H_{C\cdots M}$), which characterizes interactions with high degree of electron sharing, which in turn reflects a high degree of the C⋯M bond covalency [194]. On the other hand, the dimers with a magnesium atom feature $H_{C\cdots M}$ values close to zero.

**Figure 15:**
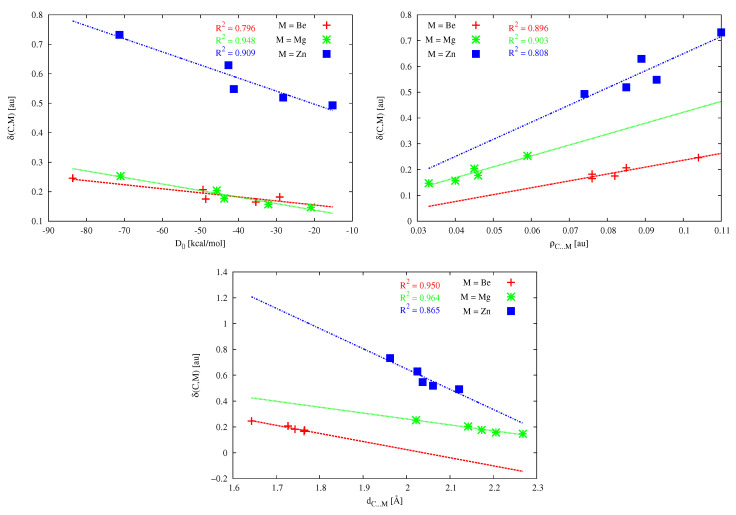
Relationships between the delocalization index of the C and M atomic basins (δ(C,M)) and the dimer dissociation energy ($D_{0}$), the electron density at the bond critical point of the C⋯M interaction ($\rho_{C\cdots M}$), or the C⋯M bond distance ($d_{C\cdots M}$) determined for the dimers in Table 5.

A very important QTAIM parameter often used to describe the A–B bond strength [195] is the so-called delocalization index δ(A,B), which defines the exchange of the electrons in the basins of atoms A and B [182,183,184]. Interestingly, particularly high values of δ(C,M) characterize the zinc bond, especially in (NH${}_{3}$)${}_{2}$C⋯ZnBr${}_{2}$ (0.732 au) and (PH${}_{3}$)${}_{2}$C⋯ZnBr${}_{2}$ (0.629 au), i.e., the systems in which CDP acts as the carbon atom donor. In a clear contrast, the δ(C,M) values for the dimers involving magnesium are similar to the δ(C,M) values for the dimers containing beryllium and are significantly lower than those for the dimers with zinc. Thus, surprisingly, the zinc bond to a carbene or CDP carbon atom should be much stronger than the corresponding magnesium or beryllium bond, of course, provided that the delocalization index is indeed a good measure of bond strength [195]. It is worth checking at this point whether there are strong linear relationships between the determined δ(C,M) values and other parameters describing the C⋯M bond strength, such as $D_{0}$, $d_{C\cdots M}$ and $\rho_{C\cdots M}$. These relationships are shown in Figure 15.

The quality of the obtained linear correlations clearly depends on both the correlated parameters and the type of the metal atom in the MX${}_{2}$ molecule. In the case of the relationship between δ(C,M) and $D_{0}$, the obtained linear correlations are reasonable for Mg and Zn ($R^{2}$ is 0.948 and 0.909, respectively), whereas the correlation for Be is rather weak ($R^{2}$ = 0.796). For the relationship between δ(C,M) and $\rho_{C\cdots M}$, the linear correlations are not great, especially for Zn ($R^{2}$ is ca. 0.9 for Be and Mg and 0.8 for Zn). In the case of the relationship δ(C,M) vs. $d_{C\cdots M}$ the $R^{2}$ values for Be and Mg are pretty good (0.950 and 0.964, respectively), whereas for Zn, the linear correlation is clearly worse ($R^{2}$ 0.865). It is worth noting that the obtained fitting lines for Zn are characterized by greater slopes, which of course results from the greater range of δ(C,M) values, from 0.493 au to 0.732 au (Table 5), thus ca. 0.24 au. In the case of Be and Mg, the range is only 0.08–0.10 au. This result indicates a greater number of electrons shared between C and Zn atomic basins than between C and either Mg or Be. Moreover, the amount is more dependent on the type of carbene or CDP.

#### 2.3.2. NCI

**Figure 16:**
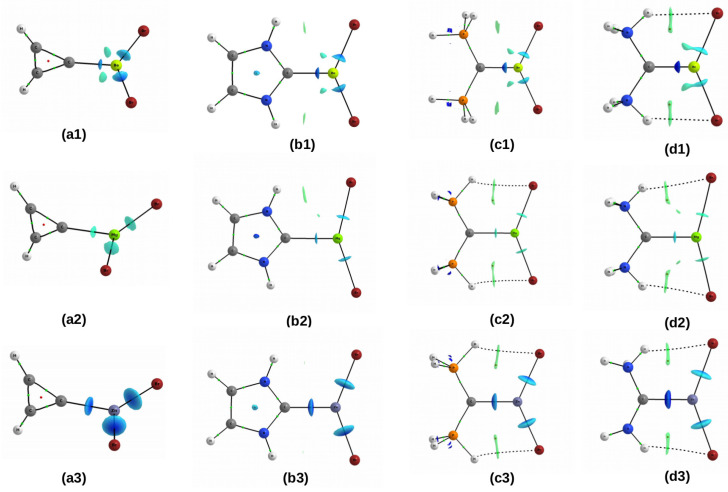
Reduced gradient density isosurfaces (*s* = 0.3 au) for dimers involving (**a**) cyclopropenylidene, (**b**) imidazol-2-ylidene, (**c**) (PH${}_{3}$)${}_{2}$C, (**d**) (NH${}_{3}$)${}_{2}$C and (**1**) BeBr${}_{2}$, (**2**) MgBr${}_{2}$, (**3**) ZnBr${}_{2}$. Colors are coded according to a common electron density scale (in au): 0.00—green, 0.05—cyan, 0.10—blue. A cutoff of 0.12 au was used for the electron density.

Most of the QTAIM-based parameters are determined at critical points (e.g., of the C⋯M bond), and therefore these parameters are local, i.e., they provide information about the properties at a particular point in space. One way out of this limitation is the NCI method [192,193], which is based on the value of the reduced electron density gradient, $s=1/\left(2{\left(3\pi^{2}\right)}^{1/3}\right)\left|\nabla \rho \right|/\rho^{4/3}$. Then, various interactions (especially those corresponding to low-density and low-gradient values) can be isolated by using appropriate cutoffs on the electron density values and its gradient. By means of the electron density gradient isosurfaces, individual interactions (especially non-covalent ones – hence the name of the method) show themselves as certain broad regions of real space rather than simply as a bond critical point between a pair of atoms [192]. In order to further investigate the difference between the zinc bond to the carbene or CDP carbon atom and its beryllium or magnesium counterpart, the electron density gradient isosurfaces were determined for the dimers of cyclopropenylidene, imidazol-2-ylidene, (PH${}_{3}$)${}_{2}$C, and (NH${}_{3}$)${}_{2}$C with MBr${}_{2}$, where M = Be, Mg and Zn (Figure 16).

The subfigure (a3) shows that the zinc bond in the cyclopropenylidene⋯ZnBr${}_{2}$ dimer does not differ significantly from both Zn-Br bonds and should be stronger than the beryllium and magnesium bonds in its counterpart dimers with BeBr${}_{2}$ (a1) and MgBr${}_{2}$ (a2), respectively. It is worth noting that in the former of these cases, i.e., in the cyclopropenylidene⋯BeBr${}_{2}$ dimer, two symmetrically located areas of weak interaction appear in the antibonding regions of the Be-Br bonds. This should lead to some elongation of both Be-Br bonds. The interaction picture in the case of imidazol-2-ylidene (b1–b3) is practically similar. In the case of the systems with MgBr${}_{2}$ (b2) and BeBr${}_{2}$, (b1) one and two regions of very weak N-H⋯Br hydrogen bonds are visible, respectively, which are *not* followed by the presence of the respective bond paths. It has been shown that the presence or absence of a bond path generally has little to do with the interaction strength [30,196,197]. Furthermore, in the case of complexes with either (PH${}_{3}$)${}_{2}$C or (NH${}_{3}$)${}_{2}$C, representing the group of carbodiphosphoranes, the characteristics of changes in the areas of weak interactions caused by the change of the Zn atom to Mg or Be are similar. Specificially, in the case of the presence of the ZnBr${}_{2}$ molecule (c3 and d3), the area for the C⋯Zn bond is similar to the area for both Zn-Br bonds, although it is distinguished by higher electron density values, especially in (NH${}_{3}$)${}_{2}$C. In the (PH${}_{3}$)${}_{2}$C⋯BeBr${}_{2}$ (c1) and (NH${}_{3}$)${}_{2}$C⋯MgBr${}_{2}$ (d2) dimers, small areas of weaker interaction develop in the antibonding zones of the metal atom and in the (NH${}_{3}$)${}_{2}$C⋯BeBr${}_{2}$ (d1) dimer, these regions merge with the regions that characterize Be-Br bonds. These interactions, however, are clearly weaker than C⋯M, especially when the metal atom is Be. It can also be seen that in all the CDP-mediated dimers, there are two symmetrical P/N-H⋯Br hydrogen bonding regions, which are or are not (case c1) followed by bond paths. However, they should be much weaker than the C⋯Zn and C⋯Be bonds.

Summing up, it can be concluded that the analysis based on the NCI method shows that the zinc bond is the strongest, and although the beryllium bond should only be slightly weaker than it, the latter is related to the presence of additional areas of weaker interaction in the antibonding regions of the Be atom. The high strength of the C⋯Zn bond (competing even with the Zn-Br bond) is reflected in high values of $\rho_{C\cdots M}$ and δ(C,M), but also in negative values of $H_{C\cdots M}$.

## 3. Theoretical Methods

Geometries of monomers and dimers were fully optimized on the ωB97X-D/6-311++G(2df,2p) level of theory, that is utilizing the ωB97X-D exchange-correlation functional [198] of Density Functional Theory (DFT) [199,200,201] and the 6-311++G(2df,2p) basis set [202,203,204,205,206], which includes both polarization and diffuse functions. By testing 200 different exchange-correlation functionals, the ωB97X-D functional has recently been shown [207] to be one of the best for general purposes. To increase the accuracy of the optimization procedure and numerical integration, cutoffs on forces and step size that are used to determine convergence were additionally tightened (0.000015 and 0.000010 for maximum force and its root mean square, respectively, and 0.000060 and 0.000040 for maximum displacement and its root mean square, respectively) and integration grid was increased to the (99, 590) one (UltraFine) having 99 radial shells and 590 angular points per shell. All the obtained systems were subjected to vibration analysis in order to check whether they correspond to the real minima on the potential energy hypersurface. There were no imaginary frequencies. Both geometry optimization and vibration analysis were performed by means of Gaussian 09 [208]. NBO-based [180,181] atomic charges were computed by means of NBO6.0 program [209] implemented in Gaussian 09. Calculations based on the QTAIM [182,183,184] and NCI [192] methods were made with the AIMAll program [210].

## 4. Conclusions

To date, the vast majority of theoretical studies on beryllium and magnesium bonds have used as Lewis bases small molecules, and the research on zinc (spodium) bonds is very rare. On the other hand, the research on carbenes and carbodiphosphoranes is mostly experimental. This article presents the results of theoretical research on the properties of beryllium, magnesium, and zinc bonds in a large group of dimers formed by the MX${}_{2}$ molecule (where M = Be, Mg, Zn and X = H, F, Cl, Br, Me) and either carbene ((NH${}_{2}$)${}_{2}$C, imidazol-2-ylidene, imidazolidin-2-ylidene, tetrahydropyrymid-2-ylidene, cyclopropenylidene) or carbodiphosphorane ((PH${}_{3}$)${}_{2}$C, (NH${}_{3}$)${}_{2}$C). Due to the rarity of theoretical studies of zinc bonds, the main focus in this article is placed on comparing them with both the beryllium bond and the magnesium bond.

The general characteristics of the presented dimers showed that the dissociation energies of the C(2)⋯M intermolecular interaction have wide range, from 10 to 53 kcal/mol, and this interaction is the strongest for the BeBr${}_{2}$ and BeCl${}_{2}$ Lewis acids. Although the C(0)⋯M bonds formed by (PH${}_{3}$)${}_{2}$C are similar in strength to the C(2)⋯M bonds formed by carbenes, (NH${}_{3}$)${}_{2}$C forms much stronger complexes, with a bond strength of up to 84 kcal/mol for the dimer with BeBr${}_{2}$. The interaction between MX${}_{2}$ and either carbene or carbodiphosphorane leads to a significant bend of the MX${}_{2}$ molecule, elongation of the MX bonds, and opening of the LCL angle (with a few exceptions).

Importantly, it has been shown that the investigated systems are characterized by very high charge transfer effect from the carbene or carbodiphosphorane molecule to the MX${}_{2}$ one. Even the weakest effect is more than twice as high as in the water dimer, while it is more than six times as strong in the (NH${}_{3}$)${}_{2}$C⋯BeBr${}_{2}$ dimer.

Theoretical studies based on the QTAIM and NCI methods have shown that the zinc bond is not very different from the beryllium bond; both should be of similar strength, while the magnesium bond should be weaker. Both are also characterized by a high degree of covalence. The determined values of the delocalization index show, however, that the zinc bond should be definitely stronger than the beryllium and magnesium bonds.

A large number of tested dimers as well as parameters characterizing both the interacting subsystems and the C⋯M bond itself allowed for the study of many linear relationships between the parameters. In general, they are good as long as systems with different M metal atoms are treated separately. The linear correlations for the zinc atom are usually slightly better than for the other atoms.

In addition to the dominant C⋯M interaction, some of the studied dimers also have various additional interactions, such as, e.g., the N-H⋯F, N-H⋯C and F-H⋯N hydrogen bonds, or N-H⋯H-Mg dihydrogen bond. In the latter case, it may be extremely short, such as 1.44 Å in (NH${}_{3}$)${}_{2}$C⋯MgH${}_{2}$. These interactions, however, are much weaker than the beryllium, magnesium, and zinc bonds that are the main topic of the research.

A side result of the presented research is that the atomic charges obtained by the QTAIM method are highly unreliable. While more reliable than these, the NBO-based atomic charges also appear to be questionable. In contrast, the Hirshfeld atomic charges appear to be chemically sound.

## Figures and Tables

**Table 1:** Some fundamental data characterizing MX${}_{2}$ molecules: the length of the M-X bond ($d_{\mathrm{MX}}$), the atomic charge (Hirshfeld-, NBO- or QTAIM-based) of M (*q*(M)), the maximum value of the electrostatic potential on M ($V^{\max}$(M)).

MX${}_{2}$	$d_{\mathrm{MX}}$/Å	*q*(M)/au			$V^{\max}$(M)/au
		Hirshfeld	NBO	QTAIM	
BeH${}_{2}$	1.335	0.421	1.185	1.689	0.057
BeF${}_{2}$	1.381	0.646	1.737	1.772	0.101
BeCl${}_{2}$	1.802	0.478	1.449	1.724	0.060
BeBr${}_{2}$	1.950	0.486	1.358	1.696	0.052
BeMe${}_{2}$	1.683	0.456	1.467	1.701	0.037
MgH${}_{2}$	1.705	0.544	1.379	1.551	0.102
MgF${}_{2}$	1.757	0.924	1.881	1.797	0.243
MgCl${}_{2}$	2.176	0.776	1.688	1.724	0.164
MgBr${}_{2}$	2.324	0.819	1.620	1.688	0.139
MgMe${}_{2}$	2.088	0.659	1.532	1.581	0.087
ZnH${}_{2}$	1.539	0.354	1.069	0.794	0.050
ZnF${}_{2}$	1.730	0.714	1.646	1.401	0.093
ZnCl${}_{2}$	2.079	0.569	1.397	1.133	0.071
ZnBr${}_{2}$	2.215	0.622	1.304	1.008	0.065
ZnMe${}_{2}$	1.942	0.444	1.284	0.861	0.037

**Table 2:** Some fundamental data characterizing carbenes and CDPs: the R-C-R angle ($\alpha_{\mathrm{RCR}}$), the atomic charge (Hirshfeld-, NBO- or QTAIM-based) of C (*q*(C) in au), the minimum value of the electrostatic potential on C ($V^{\min}$(C) in au), the energy of HOMO ($E_{\mathrm{HOMO}}$ in eV)).

Molecule	$\alpha_{\mathrm{RCR}}$	*q*(C)			$V^{\min}$(C)	$E_{\mathrm{HOMO}}$
		Hirshfeld	NBO	QTAIM		
(NH${}_{2}$)${}_{2}$C	113.0	−0.139	0.174	0.945	−0.074	−7.86
imidazol-2-ylidene	100.7	−0.188	0.104	0.787	−0.077	−8.22
imidazolidin-2-ylidene	104.7	−0.158	0.191	0.882	−0.079	−7.92
tetrahydropyrymid-2-ylidene	113.9	−0.163	0.170	0.886	−0.081	−7.41
cyclopropenylidene	55.8	−0.200	−0.108	0.040	−0.072	−8.74
(PH${}_{3}$)${}_{2}$C	127.8	−0.516	−1.536	−2.261	−0.067	−7.28
(NH${}_{3}$)${}_{2}$C	100.8	−0.563	−0.672	−0.179	−0.109	−4.35

**Table 3:** Some fundamental data characterizing carbene⋯MX${}_{2}$ dimers: C⋯M distance (in Å), changes of MX1 and MX2 bond lengths (in Å), XMX, CMX1, CMX2, LCL angles (in degrees), dissociation energy (in kcal/mol), charge transfer (in au).

R${}_{2}$C	MX${}_{2}$	$d_{C\cdots M}$	${\Delta d}_{\mathrm{MX}1}$	${\Delta d}_{\mathrm{MX}2}$	${\Delta d}_{\mathrm{MX}}^{\mathrm{av}.}$	$\alpha_{\mathrm{XMX}}$	$\alpha_{\mathrm{CMX}1}$	$\alpha_{\mathrm{CMX}2}$	$\alpha_{\mathrm{LCL}}$	${\Delta \alpha }_{\mathrm{LCL}}$	$D_{0}$	CT${}_{H}$
(NH${}_{2}$)${}_{2}$C	BeH${}_{2}$	1.820	0.048	0.048	0.048	136.4	111.8	111.8	118.9	5.9	34.7	−0.381
	BeF${}_{2}$	1.819	0.060	0.060	0.060	135.8	112.1	112.1	119.6	6.6	41.0	−0.415
	BeCl${}_{2}$	1.802	0.096	0.096	0.096	132.8	113.6	113.6	119.8	6.8	45.7	−0.394
	BeBr${}_{2}$	1.800	0.106	0.106	0.106	132.0	114.0	114.0	119.7	6.7	47.4	−0.435
	BeMe${}_{2}$	1.851	0.063	0.063	0.063	136.1	112.0	112.0	118.4	5.3	27.2	−0.381
	MgH${}_{2}$	2.268	0.064	0.033	0.049	148.5	94.2	117.3	117.7	4.6	28.2	−0.268
	MgF${}_{2}$	2.217	0.062	0.028	0.045	147.9	92.2	120.0	118.2	5.2	39.8	−0.296
	MgCl${}_{2}$	2.200	0.062	0.062	0.062	149.8	105.1	105.1	118.5	5.5	42.1	−0.320
	MgBr${}_{2}$	2.201	0.068	0.068	0.068	148.8	105.6	105.6	118.6	5.5	42.4	−0.342
	MgMe${}_{2}$	2.278	0.044	0.034	0.039	150.9	101.1	108.0	117.4	4.3	26.5	−0.301
	ZnH${}_{2}$	2.159	0.046	0.046	0.046	146.8	106.6	106.6	117.9	4.9	21.7	−0.324
	ZnF${}_{2}$	2.062	0.123	0.061	0.092	137.2	94.0	128.9	119.6	6.6	41.3	−0.364
	ZnCl${}_{2}$	2.067	0.090	0.091	0.091	141.1	109.7	109.2	119.6	6.6	40.7	−0.385
	ZnBr${}_{2}$	2.068	0.095	0.095	0.095	140.5	109.8	109.8	119.8	6.7	40.5	−0.408
	ZnMe${}_{2}$	2.202	0.054	0.054	0.054	150.8	104.6	104.6	117.6	4.5	17.1	−0.313
imidazol-2-ylidene	BeH${}_{2}$	1.793	0.048	0.048	0.048	135.3	112.4	112.4	103.5	2.7	35.4	−0.402
	BeF${}_{2}$	1.804	0.061	0.061	0.061	134.9	112.5	112.5	103.8	3.1	41.1	−0.434
	BeCl${}_{2}$	1.772	0.099	0.099	0.099	133.0	113.5	113.5	104.1	3.4	46.6	−0.427
	BeBr${}_{2}$	1.765	0.109	0.109	0.109	132.9	113.6	113.6	104.2	3.5	48.6	−0.464
	BeMe${}_{2}$	1.823	0.064	0.064	0.064	136.4	111.8	111.8	103.4	2.6	27.8	−0.401
	MgH${}_{2}$	2.246	0.073	0.030	0.051	146.3	91.7	122.0	103.0	2.2	29.2	−0.270
	MgF${}_{2}$	2.195	0.071	0.026	0.048	145.0	89.8	125.3	103.4	2.7	41.5	−0.292
	MgCl${}_{2}$	2.179	0.080	0.050	0.065	146.3	99.0	114.7	103.4	2.7	43.3	−0.335
	MgBr${}_{2}$	2.173	0.076	0.062	0.069	147.9	102.9	109.2	103.5	2.8	43.7	−0.363
	MgMe${}_{2}$	2.263	0.054	0.028	0.041	147.2	97.1	115.7	102.8	2.0	27.2	−0.302
	ZnH${}_{2}$	2.134	0.055	0.039	0.047	144.7	102.6	112.6	103.1	2.3	22.0	−0.338
	ZnF${}_{2}$	2.031	0.137	0.058	0.097	134.2	91.4	134.4	104.3	3.5	42.7	−0.365
	ZnCl${}_{2}$	2.039	0.110	0.078	0.094	138.2	104.2	117.6	104.1	3.3	41.4	−0.408
	ZnBr${}_{2}$	2.037	0.097	0.097	0.097	139.2	110.4	110.4	104.2	3.4	41.2	−0.432
	ZnMe${}_{2}$	2.179	0.061	0.050	0.056	149.2	101.3	109.5	102.9	2.1	17.2	−0.327
imidazolidin-2-ylidene	BeH${}_{2}$	1.815	0.048	0.048	0.048	135.1	112.5	112.5	107.9	3.2	34.5	−0.396
	BeF${}_{2}$	1.818	0.059	0.059	0.059	134.6	112.7	112.7	108.2	3.6	40.7	−0.428
	BeCl${}_{2}$	1.791	0.097	0.097	0.097	132.4	113.8	113.8	108.6	4.0	45.9	−0.419
	BeBr${}_{2}$	1.785	0.107	0.107	0.107	132.0	114.0	114.0	108.7	4.1	47.7	−0.457
	BeMe${}_{2}$	1.844	0.062	0.062	0.062	136.2	111.9	111.9	107.7	3.0	27.2	−0.396
	MgH${}_{2}$	2.263	0.065	0.033	0.049	146.3	94.2	119.5	107.3	2.7	28.5	−0.280
	MgF${}_{2}$	2.208	0.063	0.028	0.045	145.5	91.8	122.7	107.8	3.2	40.4	−0.306
	MgCl${}_{2}$	2.192	0.066	0.058	0.062	147.2	104.2	108.6	107.9	3.3	42.8	−0.343
	MgBr${}_{2}$	2.190	0.067	0.067	0.067	146.8	106.6	106.6	108.0	3.4	43.2	−0.361
	MgMe${}_{2}$	2.279	0.048	0.030	0.039	147.8	98.9	113.3	107.1	2.4	26.7	−0.307
	ZnH${}_{2}$	2.149	0.047	0.047	0.047	144.7	107.7	107.4	107.4	2.8	21.8	−0.339
	ZnF${}_{2}$	2.047	0.124	0.061	0.093	135.2	93.6	131.2	108.8	4.1	41.9	−0.378
	ZnCl${}_{2}$	2.052	0.092	0.092	0.092	138.4	110.8	110.8	108.7	4.0	41.4	−0.411
	ZnBr${}_{2}$	2.052	0.096	0.096	0.096	138.2	110.9	110.9	108.7	4.1	41.1	−0.428
	ZnMe${}_{2}$	2.192	0.054	0.054	0.054	149.6	105.2	105.2	107.2	2.5	17.2	−0.328
tetrahydropyrymid-2-ylidene	BeH${}_{2}$	1.806	0.052	0.052	0.052	135.5	112.2	112.2	117.9	4.0	38.9	−0.392
	BeF${}_{2}$	1.809	0.063	0.063	0.063	134.7	112.7	112.7	118.4	4.5	45.3	−0.427
	BeCl${}_{2}$	1.789	0.100	0.100	0.100	132.0	114.0	114.0	118.6	4.7	51.0	−0.408
	BeBr${}_{2}$	1.787	0.111	0.111	0.111	131.3	114.4	114.4	118.6	4.7	52.8	−0.451
	BeMe${}_{2}$	1.837	0.066	0.066	0.066	135.2	112.4	112.4	117.6	3.7	31.2	−0.393
	MgH${}_{2}$	2.250	0.069	0.036	0.053	146.8	94.6	118.6	117.2	3.3	31.9	−0.279
	MgF${}_{2}$	2.202	0.064	0.030	0.047	146.3	93.0	120.7	117.6	3.7	43.8	−0.311
	MgCl${}_{2}$	2.184	0.066	0.066	0.066	148.2	105.9	105.9	117.8	3.9	46.7	−0.336
	MgBr${}_{2}$	2.184	0.072	0.072	0.072	147.4	106.3	106.3	117.9	4.0	47.2	−0.359
	MgMe${}_{2}$	2.258	0.048	0.037	0.042	149.2	101.8	109.0	116.9	3.0	29.9	−0.314
	ZnH${}_{2}$	2.135	0.050	0.050	0.050	145.0	107.5	107.6	117.4	3.5	25.5	−0.340
	ZnF${}_{2}$	2.044	0.129	0.064	0.096	134.7	94.6	130.6	118.7	4.8	46.3	−0.384
	ZnCl${}_{2}$	2.049	0.096	0.096	0.096	139.3	110.3	110.4	118.6	4.8	46.2	−0.407
	ZnBr${}_{2}$	2.050	0.100	0.100	0.100	138.9	110.5	110.5	118.7	4.8	45.9	−0.428
	ZnMe${}_{2}$	2.173	0.059	0.059	0.059	148.8	105.6	105.6	117.1	3.2	20.6	−0.332
cyclopropenylidene	BeH${}_{2}$	1.743	0.039	0.039	0.039	135.1	112.4	112.4	56.6	0.8	29.1	−0.317
	BeF${}_{2}$	1.833	0.050	0.050	0.050	135.9	112.0	112.0	57.2	1.4	29.4	−0.386
	BeCl${}_{2}$	1.781	0.084	0.084	0.084	134.3	112.8	112.8	57.3	1.5	33.6	−0.389
	BeBr${}_{2}$	1.764	0.093	0.093	0.093	134.5	112.8	112.8	57.3	1.5	35.4	−0.414
	BeMe${}_{2}$	1.784	0.061	0.061	0.061	135.2	112.4	112.4	56.7	0.9	20.2	−0.329
	MgH${}_{2}$	2.268	0.035	0.035	0.035	148.3	105.9	105.9	56.7	0.9	20.9	−0.273
	MgF${}_{2}$	2.222	0.031	0.031	0.031	147.9	106.1	106.1	56.9	1.2	29.3	−0.325
	MgCl${}_{2}$	2.210	0.047	0.047	0.047	145.6	107.2	107.2	57.0	1.2	32.0	−0.323
	MgBr${}_{2}$	2.206	0.052	0.052	0.052	145.4	107.3	107.3	57.0	1.3	32.1	−0.331
	MgMe${}_{2}$	2.288	0.030	0.030	0.030	149.1	105.4	105.4	56.6	0.8	19.4	−0.275
	ZnH${}_{2}$	2.121	0.036	0.036	0.036	144.6	107.7	107.7	57.3	0.9	15.2	−0.279
	ZnF${}_{2}$	2.068	0.072	0.072	0.072	138.7	110.6	110.6	57.3	1.5	28.7	−0.378
	ZnCl${}_{2}$	2.063	0.078	0.078	0.078	137.5	111.3	111.3	57.3	1.5	28.6	−0.371
	ZnBr${}_{2}$	2.061	0.083	0.083	0.083	137.1	111.5	111.5	57.3	1.5	28.2	−0.380
	ZnMe${}_{2}$	2.192	0.048	0.048	0.048	149.0	105.5	105.5	56.6	0.8	10.3	−0.270

**Table 4:** Some fundamental data characterizing CDP⋯MX${}_{2}$ dimers: C⋯M distance (in Å), changes of MX1 and MX2 bond lengths (in Å), XMX, CMX1, CMX2, LCL angles (in degrees), dissociation energy (in kcal/mol), charge transfer (in au).

R${}_{2}$C	MX${}_{2}$	$d_{C\cdots M}$	${\Delta d}_{\mathrm{MX}1}$	${\Delta d}_{\mathrm{MX}2}$	${\Delta d}_{\mathrm{MX}}^{\mathrm{av}.}$	$\alpha_{\mathrm{XMX}}$	$\alpha_{\mathrm{CMX}1}$	$\alpha_{\mathrm{CMX}2}$	$\alpha_{\mathrm{LCL}}$	${\Delta \alpha }_{\mathrm{LCL}}$	$D_{0}$	CT${}_{H}$
(PH${}_{3}$)${}_{2}$C	BeH${}_{2}$	1.767	0.058	0.058	0.058	132.9	113.5	113.5	131.3	3.5	35.5	−0.354
	BeF${}_{2}$	1.792	0.071	0.071	0.071	130.9	114.5	114.5	128.9	1.2	40.4	−0.407
	BeCl${}_{2}$	1.736	0.120	0.120	0.120	129.1	115.5	115.5	126.9	−0.9	47.0	−0.394
	BeBr${}_{2}$	1.727	0.133	0.133	0.133	128.3	115.9	115.9	125.4	−2.4	49.3	−0.455
	BeMe${}_{2}$	1.811	0.077	0.077	0.077	130.3	114.8	114.8	128.3	0.5	26.2	−0.361
	MgH${}_{2}$	2.199	0.054	0.054	0.054	147.5	106.2	106.2	127.8	0.1	30.7	−0.259
	MgF${}_{2}$	2.146	0.048	0.048	0.048	147.6	106.2	106.2	129.1	1.3	41.7	−0.326
	MgCl${}_{2}$	2.143	0.074	0.074	0.074	139.8	110.1	110.1	124.1	−3.7	45.3	−0.315
	MgBr${}_{2}$	2.142	0.082	0.082	0.082	137.9	111.0	111.0	123.1	−4.7	45.7	−0.354
	MgMe${}_{2}$	2.226	0.046	0.046	0.046	143.2	108.4	108.4	126.6	−1.2	28.4	−0.281
	ZnH${}_{2}$	2.128	0.050	0.050	0.050	146.7	106.7	106.7	127.3	−0.4	22.7	−0.271
	ZnF${}_{2}$	2.026	0.094	0.094	0.094	140.1	110.0	110.0	129.8	2.0	42.7	−0.384
	ZnCl${}_{2}$	2.025	0.107	0.107	0.107	133.1	113.5	113.5	125.4	−2.3	43.0	−0.367
	ZnBr${}_{2}$	2.025	0.112	0.112	0.112	131.3	114.4	114.4	124.6	−3.2	42.6	−0.409
	ZnMe${}_{2}$	2.182	0.060	0.060	0.060	143.9	108.1	108.1	125.0	−2.7	17.0	−0.265
(NH${}_{3}$)${}_{2}$C	BeH${}_{2}$	1.655	0.118	0.060	0.089	133.2	103.1	123.8	111.6	10.8	64.0	−0.527
	BeF${}_{2}$	1.718	0.118	0.074	0.096	129.9	107.5	122.3	109.5	8.7	67.0	−0.520
	BeCl${}_{2}$	1.661	0.149	0.149	0.149	132.3	113.7	113.7	111.6	10.8	79.3	−0.539
	BeBr${}_{2}$	1.643	0.167	0.167	0.167	132.9	113.5	113.5	112.4	11.5	83.6	−0.610
	BeMe${}_{2}$	1.695	0.097	0.097	0.097	126.9	116.5	116.5	109.5	8.7	52.5	−0.524
	MgH${}_{2}$	2.076	0.172	0.041	0.106	136.8	86.6	136.6	108.1	7.3	54.6	−0.329
	MgF${}_{2}$	2.064	0.133	0.039	0.086	135.4	89.3	135.4	108.3	7.5	67.6	−0.388
	MgCl${}_{2}$	2.044	0.152	0.072	0.112	140.6	97.0	122.5	108.7	7.9	70.0	−0.465
	MgBr${}_{2}$	2.022	0.122	0.122	0.122	152.0	104.0	104.0	110.6	9.8	71.0	−0.513
	MgMe${}_{2}$	2.086	0.154	0.037	0.096	128.8	97.3	133.9	108.0	7.2	48.6	−0.377
	ZnH${}_{2}$	1.979	0.161	0.035	0.098	133.1	91.7	135.1	108.7	7.9	45.1	−0.427
	ZnF${}_{2}$	1.879	0.660	0.040	0.350	109.8	82.1	167.5	110.9	10.0	78.8	−0.287
	ZnCl${}_{2}$	1.952	0.212	0.091	0.151	130.1	99.7	130.1	108.8	8.0	71.1	−0.535
	ZnBr${}_{2}$	1.962	0.142	0.142	0.142	139.8	109.8	109.8	109.2	8.4	71.3	−0.569
	ZnMe${}_{2}$	2.021	0.087	0.087	0.087	136.7	111.7	111.7	107.3	6.5	36.3	−0.481

**Table 5:** Some fundamental QTAIM-based parameters (in au) characterizing carbene⋯MX${}_{2}$ and CDP⋯MX${}_{2}$ dimers: the electron density ($\rho_{C\cdots M}$), its Laplacian ($\nabla^{2}\rho_{C\cdots M}$) and the total electronic energy density ($H_{C\cdots M}$) computed at the C⋯M bond critical point, the delocalization index of the C and M atomic basins (δ(C,M)).

Dimer	$\rho_{C\cdots M}$	${\nabla^{2}\rho }_{C\cdots M}$	$H_{C\cdots M}$	δ(C,M)
cyclopropenylidene⋯BeH${}_{2}$	0.076	0.323	−0.022	0.182
cyclopropenylidene⋯MgH${}_{2}$	0.033	0.163	0.003	0.147
cyclopropenylidene⋯ZnH${}_{2}$	0.074	0.205	−0.023	0.493
cyclopropenylidene⋯BeBr${}_{2}$	0.076	0.288	−0.025	0.165
cyclopropenylidene⋯MgBr${}_{2}$	0.040	0.194	0.001	0.157
cyclopropenylidene⋯ZnBr${}_{2}$	0.085	0.236	−0.028	0.519
imidazol-2-ylidene⋯BeBr${}_{2}$	0.082	0.272	−0.031	0.175
imidazol-2-ylidene⋯MgBr${}_{2}$	0.046	0.218	0.000	0.177
imidazol-2-ylidene⋯ZnBr${}_{2}$	0.093	0.249	−0.032	0.548
(PH${}_{3}$)${}_{2}$C⋯BeBr${}_{2}$	0.085	0.259	−0.036	0.207
(PH${}_{3}$)${}_{2}$C⋯MgBr${}_{2}$	0.045	0.208	−0.001	0.204
(PH${}_{3}$)${}_{2}$C⋯ZnBr${}_{2}$	0.089	0.209	−0.032	0.629
(NH${}_{3}$)${}_{2}$C⋯BeBr${}_{2}$	0.104	0.361	−0.047	0.246
(NH${}_{3}$)${}_{2}$C⋯MgBr${}_{2}$	0.059	0.309	−0.003	0.253
(NH${}_{3}$)${}_{2}$C⋯ZnBr${}_{2}$	0.110	0.276	−0.041	0.732

## Sample Availability

Optimized geometries of all considered systems are available from the author on request.

## Abbreviations

The following abbreviations are used in this manuscript:

CDPs	Carbodiphosphoranes
MESP	Molecular electrostatic potential
NBO	Natural Bond Orbital
QTAIM	Quantum Theory of Atoms in Molecules
NCI	Noncovalent Interactions (method)
